# Lobectomy Increases Postoperative Pulmonary Artery Enlargement to a Greater Extent than Segmentectomy

**DOI:** 10.5761/atcs.oa.24-00083

**Published:** 2025-01-28

**Authors:** Megumi Nishikubo, Yugo Tanaka, Shinya Tane, Daisuke Hokka, Yoshimasa Maniwa

**Affiliations:** Division of Thoracic Surgery, Department of Surgery, Kobe University Hospital and Graduate School of Medicine, Kobe, Hyogo, Japan

**Keywords:** lung cancer, segmentectomy, pulmonary artery, right ventricular function, computed tomography

## Abstract

**Purpose:** The underlying mechanism why segmentectomy has demonstrated the non-inferiority to lobectomy in several randomized trials remains unclear. Computed tomography (CT)-measured pulmonary artery (PA) enlargement reflects PA pressure and predicts the prognosis of certain respiratory diseases. We compared the preoperative and postoperative PA diameter to the ascending aorta diameter (PA/A) ratio, investigating its impact on right ventricular function in lung resection.

**Methods:** This retrospective study was conducted in patients with lower-lobe lung tumors who underwent anatomical lung resection between 2017 and 2022. The PA diameter at the bifurcation and the ascending aorta diameter at the same CT image slice were measured preoperatively and postoperatively. We calculated the enlargement of PA/A ratio (PA/A change) and compared lobectomy and segmentectomy.

**Results:** This analysis included 279 patients (235 with lobectomy and 44 with segmentectomy). The PA/A change was significantly greater in patients with lobectomy than segmentectomy (104% vs. 102%, P = 0.02). In the multivariable analysis, airflow obstruction (yes, P = 0.04) and the type of surgery (segmentectomy, P = 0.04) were independent prognostic factors for PA/A change.

**Conclusions:** The PA/A change was greater in lobectomy than in segmentectomy. This change could reflect a burden on right ventricular function after lobectomy.

## Introduction

Lobectomy with complete lymph node dissection has been the gold standard of treatment for early-stage lung cancer, as reported by a 1995 randomized controlled study.^1)^ However, several recent multicenter randomized trials have shown the non-inferiority of limited resection, such as segmentectomy or wedge resection, for small-sized non-small-cell lung cancer (NSCLC).^2–4)^

The randomized controlled trial, JCOG0802, showed long overall survival (OS) with segmentectomy despite short relapse-free survival in patients with small-sized peripheral NSCLC. However, the median reduction of the forced expiratory volume in 1 s (FEV1) was only 3% between lobectomy and segmentectomy. This finding was statistically significant, but the relationship between the difference in FEV1 and a short OS is unclear. Some kind of factor to shorten the OS of lobectomy is suggested.

Lung resection causes lung volume loss and affects hemodynamics, especially right ventricle (RV) functioning.^5–7)^ Deslauriers is often quoted as saying, “Pneumonectomy is a disease in itself.” Smulders has reported RV dysfunction even after 5 years of pneumonectomy.^8)^ However, the long-term effects of other procedures, including lobectomy or segmentectomy, on RV function are unclear. Moreover, the difference between lobectomy and segmentectomy has not been investigated.

In 2014, Iyer reported a linear correlation between computed tomography (CT)-measured pulmonary artery (PA) enlargement, defined as a PA to ascending aorta (A) diameter (PA/A) ratio, and mean PA pressure (PAP). This finding was not observed with PA systolic pressure (PASP) measured via echocardiography.^9)^ Several reports measuring various risks using the PA/A ratio were published. Wells^10)^ showed that a PA/A ratio >1 was associated with severe chronic obstructive pulmonary disease (COPD) exacerbations. Asakura^11)^ demonstrated that the PA/A ratio could predict postoperative cardiopulmonary complications after lung cancer surgery.

Hence, we hypothesized that postoperative RV dysfunction differs after lobectomy and segmentectomy. To confirm this hypothesis, we compared the change in PA enlargement pre- and post-lobectomy and segmentectomy using the PA/A ratio.

## Materials and Methods

### Study design and patient selection

Records of patients who underwent lobectomy or segmentectomy for lower-lobe tumors, between January 2017 and December 2022, at Kobe University Hospital were analyzed. Primary lung cancer, metastatic lung tumors, malignant lymphoma, and benign tumors were included. Patients with ≥20 years of age and a CT scan performed before and 3–12 months post-surgery were eligible. Cases with a history of thoracic surgery, aortic aneurysm, combined resection of neighboring organs, bilobectomy, and simultaneous tumor resection of another lobe were excluded.

The protocol complied with the Declaration of Helsinki and was approved by the Clinical Research Area Ethics Committee of Kobe University Graduate School of Medicine on October 26, 2023 (Registration no.: B230138). Written informed consent regarding the research use of their data was obtained as an opt-out from each patient.

### Surgical procedure

Standard pulmonary resection was conducted using video-assisted thoracic surgery (VATS) or robotic-assisted thoracoscopic surgery (RATS). In some cases, the thoracotomy approach was used because bronchoplasty or PA plasty was required. Complete VATS was conducted with 4 port-incisions, with a maximum size of 2.0–3.0 cm. RATS was performed with a da Vinci Surgical System (Intuitive Surgical, Sunnyvale, CA, USA).

### Clinical characterization

Clinical and laboratory data were collected from medical records at Kobe University Hospital. Airflow obstruction was defined as a FEV1 to a total forced vital capacity ratio (FEV1/FVC) of <0.7. Pulmonary hypertension was assessed via transthoracic echocardiography in a limited number of patients, defined as a peak tricuspid regurgitation (TR) velocity of >2.8 m/s.^12)^ Postoperative cardiopulmonary complications included pneumonia, respiratory failure, arrhythmias, and acute heart failure.

### Measuring the PA/A change

**Fig. 1:**
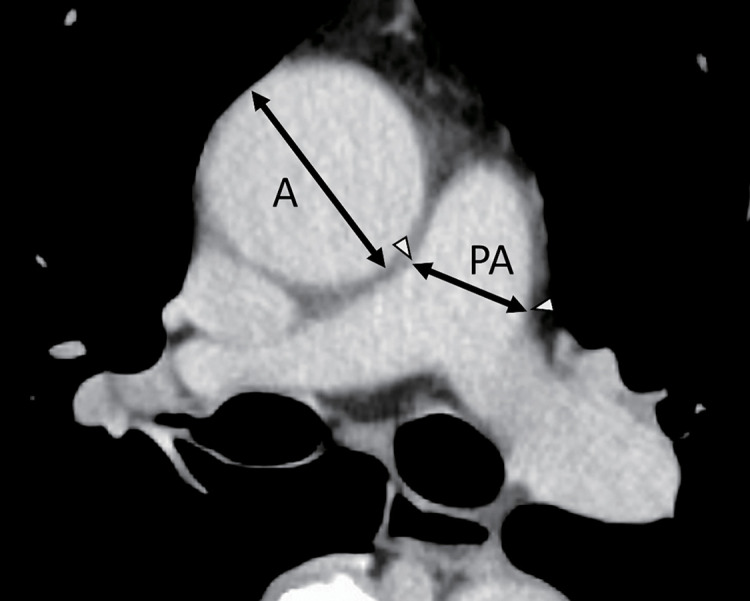
PA and aorta (**A**) diameter measurements; chest CT axial image was used, and the measuring point of the main PA is the PA bifurcation; ascending aorta diameter was measured at the same CT slice as the PA measurement; red arrowheads show the bifurcation of the bilateral pulmonary arteries, and black arrows show the PA and A diameter to calculate the PA/A ratio. A: aorta; CT: computed tomography; PA: pulmonary artery; PA/A ratio: pulmonary artery to aorta diameter ratio

One reviewer analyzed axial CT scans and measured the primary PA diameter at the PA bifurcation level. The ascending aorta diameter was measured at the same slice level as the PA measurements. According to a previous report,^11)^ the measuring point of the primary PA is at the bifurcation of the PA because these points are obvious on an axial CT in most patients and have little error (**Fig. 1**).

We calculated the PA/A ratio using CT scans conducted before and after surgery (pre- and post-PA/A ratio). The post-PA/A ratio value was divided by the pre-PA/A ratio and defined as the “PA/A change.” To compare the incidence of postoperative cardiopulmonary complications, we categorized the patients into 2 groups based on the change in PA/A, ensuring that both the high and low PA/A change groups contained an equal number of patients.

### Statistical analysis

Baseline data were expressed as medians and percentages for continuous and categorical variables, respectively. We compared the PA/A change between lobectomy and segmentectomy; continuous variables were compared using the 2-sided Wilcoxon rank-sum test. Categorical variables were analyzed using the χ^2^ test. A multiple regression analysis was used for multivariable analysis. Age, comorbidities, cardiovascular risks, and lung functions are crucial in the physiological evaluation of a patient considered for lung resection.^13)^ Therefore, age, airflow obstruction, cardiovascular comorbidity (coronary artery disease, congestive heart failure, or arrhythmia), and the type of surgical procedure were explanatory variables in the multiple regression analysis. All statistical analyses were performed using R (R Foundation for Statistical Computing, Vienna, Austria). Significance was determined to be P <0.05.

## Results

**Fig. 2:**
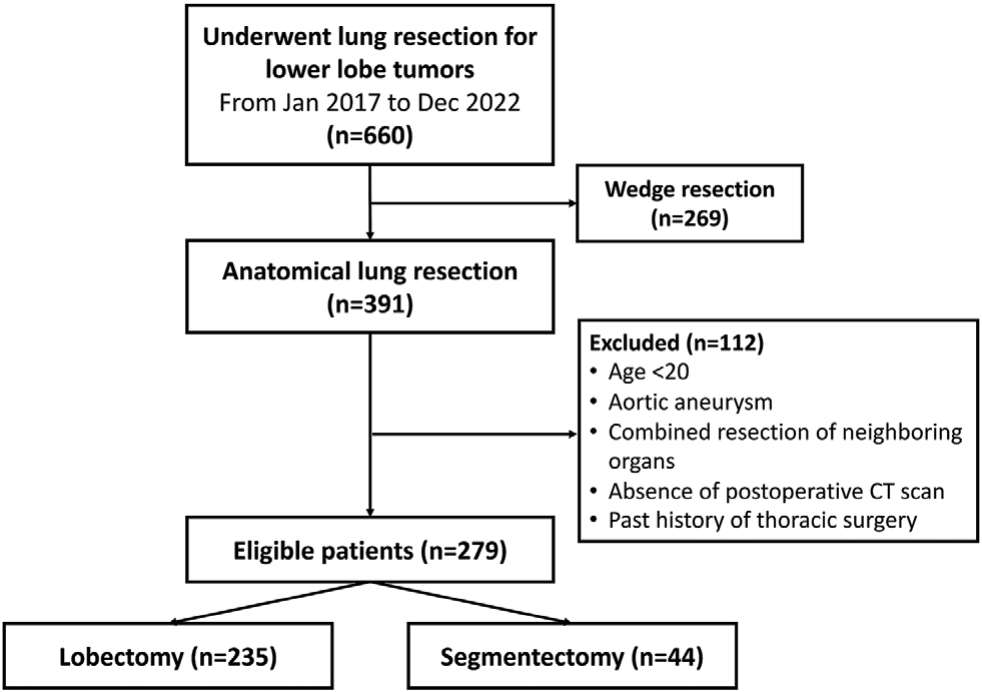
Study cohort selection flowchart. CT: computed tomography

In total, 391 patients underwent lobectomy or segmentectomy for lower-lobe tumors between January 2017 and December 2022. Among them, 279 patients were included in our study cohort (**Fig. 2**).

**Fig. 3:**
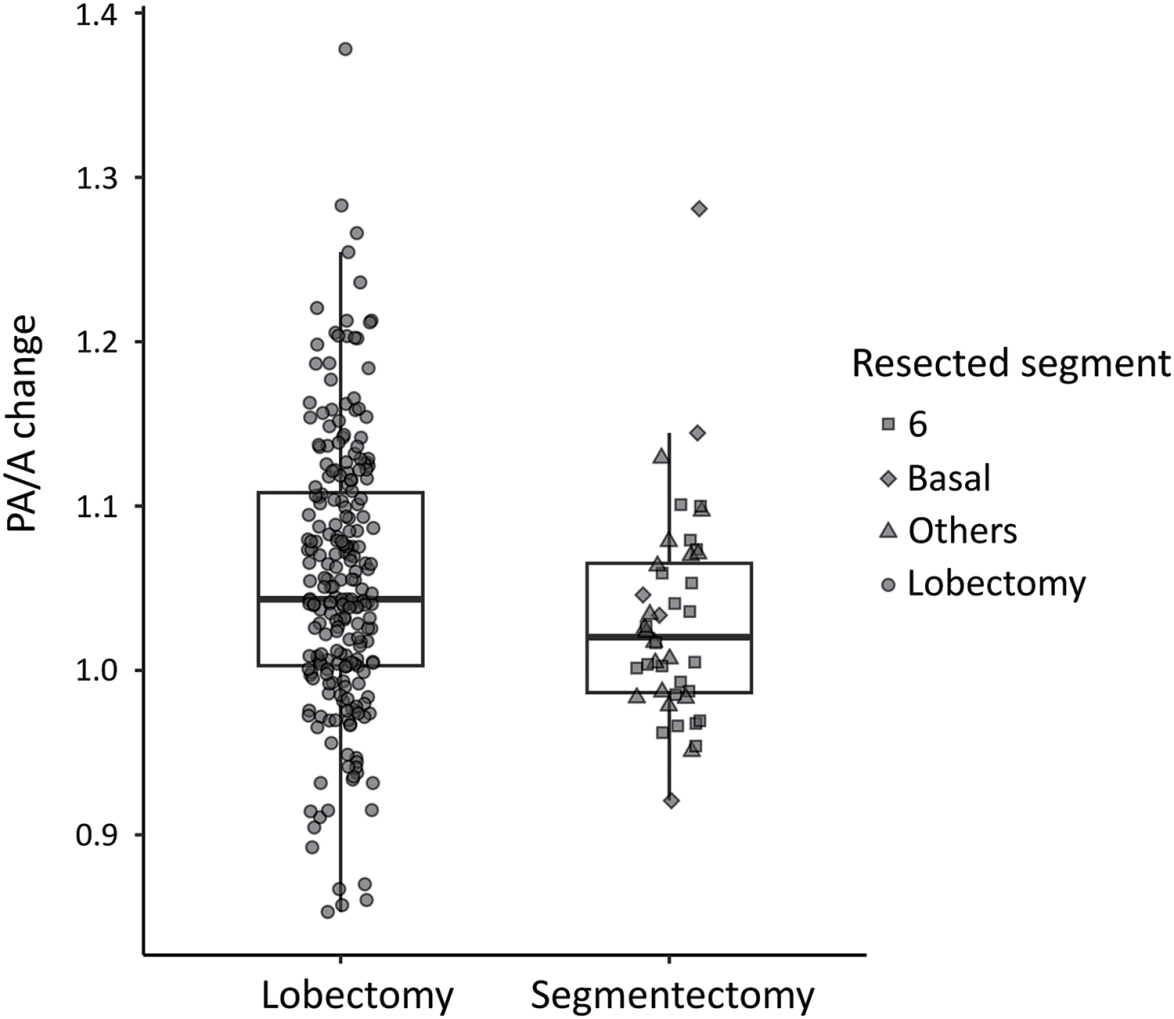
Box plot and scatter plot showing the PA to ascending aorta diameter ratio change (PA/A change); PA/A change was significantly greater in lobectomy than segmentectomy patients. PA: pulmonary artery; PA/A change: PA to ascending aorta diameter ratio change

**Table 1** summarizes the clinical characteristics of the patients; the age, sex, side of the tumors, airflow obstruction, and the rate of primary lung cancer did not differ significantly between the 2 groups. A total of 100 patients underwent echocardiography as a preoperative assessment, of whom 2 were identified as having pulmonary hypertension. Additionally, among those who underwent preoperative echocardiography, 21 patients underwent echocardiography postoperatively and measured TR velocity. The median preoperative and postoperative PA and aorta diameters are shown in **Table 1**. The median preoperative PA/A ratio was 0.75 and 0.70 in lobectomy and segmentectomy groups, respectively (P = 0.12); the median postoperative PA/A ratio was 0.78 and 0.74, respectively (P = 0.02), and the PA/A change was significantly higher in lobectomy than segmentectomy (104% vs. 102%, P = 0.02) (**Fig. 3**). Similarly, the median change rate of preoperative and postoperative TR velocity tend to be higher in lobectomy than segmentectomy, though it is not significant (111% vs. 109%, P = 0.89). The detailed segmentectomy surgical procedure is shown in **Table 1**. Additionally, the relationship between the precise segmentectomy procedure and PA/A change appeared unrelated (**Fig. 3**).

**Table 1:** Clinicopathological characteristics

Characteristics	Lobectomy (n = 235)	Segmentectomy (n = 44)	P
Median age, years (IQR)	72.0 (66.0, 76.0)	71.0 (65.5, 75.0)	0.32
Sex			0.92
Male	139 (59.1)	27 (61.4)	
Female	96 (40.9)	17 (38.6)	
Median preoperative PA diameter, mm (IQR)	26.1 (24.0, 28.7)	24.7 (22.0, 28.4)	0.08
Median postoperative PA diameter, mm (IQR)	27.5 (25.1, 30.5)	25.8 (23.6, 28.8)	0.01
Median preoperative A diameter, mm (IQR)	35.2 (32.7, 37.9)	35.3 (33.6, 36.7)	0.80
Median postoperative A diameter, mm (IQR)	35.1 (32.8, 37.8)	35.5 (33.3, 37.4)	0.96
Median preoperative PA/A ratio (IQR)	0.75 (0.69, 0.83)	0.70 (0.66, 0.82)	0.12
Median postoperative PA/A ratio (IQR)	0.78 (0.72, 0.88)	0.74 (0.67, 0.82)	0.02
Median PA/A change (IQR)	1.04 (1.00, 1.11)	1.02 (0.99, 1.07)	0.02
Preoperative PH detected via echocardiography			NA
Yes	2 (0.9)	0 (0.0)	
No	80 (34.0)	18 (40.9)	
Did not undergo echocardiography	153 (65.1)	26 (59.1)	
Median TR velocity change (IQR)	1.11 (0.92, 1.22)	1.09 (1.03, 1.16)	0.89
Tumor location			0.72
Right	133 (56.6)	23 (52.3)	
Left	102 (43.4)	21 (47.7)	
Airflow obstruction			0.73
Yes	76 (32.3)	16 (36.4)	
No	159 (67.7)	28 (63.6)	
Disease			0.09
Lung cancer	213 (90.6)	36 (81.8)	
Metastatic tumor	19 (8.1)	8 (18.2)	
Others	3 (1.3)	0 (0.0)	
Segment			NA
6	NA	21 (47.7)	
8	NA	8 (18.2)	
Basal	NA	5 (11.4)	
9 + 10	NA	5 (11.4)	
8 + 9	NA	2 (4.5)	
6 + 10	NA	1 (2.3)	
8a	NA	1 (2.3)	
10	NA	1 (2.3)	

Values are presented as n (%) unless otherwise indicated.

A: aorta; IQR: interquartile range; NA: not available; PA: pulmonary artery; PA/A ratio: pulmonary artery to aorta diameter ratio; PH: pulmonary hypertension; TR: tricuspid regurgitation

In the multivariable analysis, airflow obstruction (yes, estimate value 0.02, 95% CI, 0.0009 to 0.04, P = 0.04) and the type of surgery (segmentectomy, estimate value −0.03, 95% CI, −0.05 to −0.002, P = 0.04) were independent prognostic factors for the PA/A change (**Table 2**).

**Table 2:** Multiple regression analysis for the prediction of PA/A change

Variable	Estimate (95% CI)	Standard error	t	P
Age	−0.000007 (−0.001, 0.001)	0.00060	−0.01	>0.99
Airflow obstruction (yes)	0.021 (0.0009, 0.041)	0.010	2.06	0.04
Type of surgery (segmentectomy)	−0.028 (−0.054, −0.002)	0.013	−2.11	0.04
Cardiovascular comorbidity (yes)	0.012 (−0.014, 0.039)	0.014	0.91	0.37

CI: confidence interval; PA/A change: pulmonary artery to ascending aorta diameter ratio change

As for the relationship between PA/A change and the incidence of postoperative cardiopulmonary complications, the incidence was higher in the high PA/A change group than in the low PA/A change group, although this difference was not significant (6.4% vs. 3.6%, P = 0.42).

## Discussion

We demonstrated that the PA/A change differs between lobectomy and segmentectomy, and airflow obstruction and the type of surgery were independent factors for the PA/A change. This result could help elucidate the influence of physical burden and the prognosis of lung resection.

First, the PA/A change was greater after lobectomy than after segmentectomy. Iyer^9)^ showed that the CT-measured PA/A ratio correlates with PAP determined by right-sided heart catheterization (RHC) in patients with severe COPD, and this finding was more robust than PASP measured via echocardiography. Our results state that the PA/A change differs between lobectomy and segmentectomy implying that lobectomy increases PAP to a greater extent than segmentectomy, resulting in RV dysfunction. Furthermore, the median change rate of preoperative and postoperative TR velocity tends to be higher in lobectomy than in segmentectomy among the limited number of patients who had TR velocity measured preoperatively and postoperatively. Although this difference is not statistically significant due to the small sample size, it is consistent with the main outcome regarding PA/A change.

Asakura^11)^ reported that postoperative cardiopulmonary complications were significantly higher in patients with PA/A ratios >1.0 than in those with PA/A ratios ≤1.0; they concluded that this difference is caused by pulmonary hypertension. Pulmonary resection causes a loss of the PA bed. Several studies have reported RV pump performance and PAP deterioration after major pulmonary resection.^5,6)^ Researchers speculated that it was due to the change in the RV afterload. Furthermore, RV dysfunction is more pronounced in pneumonectomy than in lobectomy.^14)^ Although no report has compared the RV dysfunction between lobectomy and segmentectomy, lobectomy causes a greater lung volume loss than segmentectomy. Hence, it could cause more RV dysfunction than expected by lung function. One concern is the minimal differences in the PA/A change and the PA/A ratio between segmentectomy and lobectomy. Nonetheless, in previous studies, the odds ratio for postoperative cardiopulmonary complications was 2.3 for every 0.1-point increase in PA/A ratio, and the major complication and 30-day mortality odds for every 1 mm enlargement of PA diameter were 1.12 and 1.25, respectively.^11,15)^ Considering these findings, the differences observed in our study were substantial. An analysis of OS will be more informative in elucidating the clinical influence of PA enlargement on the patients. However, this study included patients with metastatic tumors and the follow-up period was too short to calculate OS. Further studies are required to examine the actual clinical implications of the observed differences.

Next, airflow obstruction and the type of surgery were independent predictors of the PA/A change in multivariable analysis. Patients with COPD have a higher risk of postoperative complications, considerably due to emphysematous changes and low lung function.^16)^ However, regarding our result, surgery could have a profound influence on RV dysfunction in patients with COPD.

This study had several limitations. First, it is retrospective, and we could not remove unknown confounding factors. Second, we could not analyze the relationship between the PA/A ratio and PAP. Pulmonary hypertension was assessed via transthoracic echocardiography in a limited number of patients, and we found similar results for TR velocity as we did for the PA/A ratio. However, RHC was not performed in any patient in this study, considering it is too invasive to perform routinely; therefore, what should be used as a gold standard to measure pulmonary hypertension and RV function is a big question. Last, we only included lower-lobe tumors in this study. The main reason was that in the case of upper lobe lobectomies or segmentectomies, PA bifurcations were deformed making it difficult to compare preoperative and postoperative diameters. Thus, our results may not be generalizable to all lobectomies.

## Conclusion

CT-measured PA/A changes are larger in lobectomy than in segmentectomy. This finding could reflect the burden of lobectomy on RV function. Further investigation is required to clarify the clinical significance of the PA/A ratio in pulmonary resection, specifically analyzing which patients should avoid lobectomy to prevent postoperative complications.

## Declarations

### Ethics approval and consent to participate

The protocol complied with the Declaration of Helsinki and was approved by the Clinical Research Area Ethics Committee of Kobe University Graduate School of Medicine on October 26, 2023 (Registration No.: B230138).

### Consent for publication

Written informed consent regarding the use of patients’ data was obtained as an opt-out from each patient.

### Funding

The authors have no funding sources to disclose.

### Data availability

Not applicable.

### Author contributions

Megumi Nishikubo designed this study and analyzed the data. Megumi Nishikubo and Yugo Tanaka prepared the figures and wrote the original draft. Shinya Tane, Daisuke Hokka, and Yoshimasa Maniwa oversaw the study and revised the article. All authors reviewed and approved the final manuscript.

### Disclosure statement

The authors have no conflict of interest to disclose.
